# Induced Expression of *AtLEC1* and *AtLEC2* Differentially Promotes Somatic Embryogenesis in Transgenic Tobacco Plants

**DOI:** 10.1371/journal.pone.0071714

**Published:** 2013-08-12

**Authors:** Fengdan Guo, Chuanliang Liu, Han Xia, Yuping Bi, Chuanzhi Zhao, Shuzhen Zhao, Lei Hou, Fuguang Li, Xingjun Wang

**Affiliations:** 1 High-Tech Research Center, Shandong Academy of Agricultural Sciences, Shandong Provincial Key Laboratory of Crop Genetic Improvement, Ecology and Physiology, Jinan, PR China; 2 Cotton Research Institute of CAAS, State Key Laboratory of Cotton Biology, Anyang, PR China; 3 College of Life Sciences, Shandong Normal University, Jinan, PR China; Wuhan University, China

## Abstract

*Arabidopsis LEAFY COTYLEDON* (*LEC*) genes, *AtLEC1* and *AtLEC2*, are important embryonic regulators that play key roles in morphogenesis and maturation phases during embryo development. Ectopic expression of *AtLEC1* and *AtLEC2* in tobacco caused abnormality in transgenic seedling. When transgenic seeds germinated on medium containing 30 µM DEX, *LEC1* transgenic seedlings were ivory and fleshy, with unexpanded cotyledons, stubby hypocotyls, short roots and no obvious callus formation at the shoot meristem position. While *LEC2* transgenic seedlings formed embryonic callus on the shoot apical meristem and somatic embryo-like structures emerged from the surface of the callus. When callus were transferred to hormone free MS_0_ medium more shoots were regenerated from each callus. However, shoot formation was not observed in *LEC1* overexpressors. To investigate the mechanisms of *LEC2* in somatic embryogenesis, we studied global gene expression by digital gene expression profiling analysis. The results indicated that ectopic expression of *LEC2* genes induced accumulation of embryo-specific proteins such as seed storage proteins, late embryogenesis abundant (LEA) proteins, fatty acid biosynthetic enzymes, products of steroid biosynthesis related genes and key regulatory genes of the embryo development. Genes of plant-specific transcription factors such as NAC domain protein, *AP2* and *GRAS* family, resistance-related as well as salicylic acid signaling related genes were up-regulated in *LEC2* transgenic seedlings. Ectopi c expression of *LEC2* induced large number of somatic embryo formation and shoot regeneration but 20 d DEX induction of *LEC1* is not sufficient to induce somatic embryogenesis and shoot formation. Our data provide new information to understand the mechanisms on *LEC2* gene’s induction of somatic embryogenesis.

## Introduction

In higher plants, embryogenesis is a key developmental event under precise genetic regulation. After fertilization, the fertilized egg undergoes a series of biological process, such as zygote activation, polarity establishment, pattern formation and organogenesis. Subsequently, developing embryos enter maturation phase, during which storage reserves are accumulated abundantly and embryos acquire desiccation tolerance. Finally, the mature and dormant seeds with a quiescent metabolic embryo are formed. Somatic embryogenesis is that somatic cells, under inductive conditions, undergo a series of biological process to generate somatic embryos [1]. The somatic embryos undergo processes closely resemble that of zygotic embryogenesis. Somatic embryogenesis provides a model system for studying molecular and biochemical mechanism of zygotic embryogenesis.

Many crops exhibit low efficiency of regeneration, which may negatively affect the progress of yield, quality or stress tolerance improvement by genetic modification. Increasing the regeneration rate of crops through either somatic embryogenesis or organogenesis and establishment of high efficient plant regenerating system is a key step for gene engineering improvement of crops such as soybean, cotton and peanut. *Leafy Cotyledon* (*LEC*) genes including *LEC1* and *LEC2* are key regulators of plant embryo development. They play key roles during both embryo morphogenesis and maturation phases. Both *lec1* and *lec2* mutant embryos are known to show trichomes on the cotyledons, lack of embryo-specific proteins and loss of desiccation tolerance [2], [3].

*LEC1* encodes HAP3 subunit of CCAAT-binding transcription factor. Ectopic expression of *LEC1* gene was sufficient to confer transgenic seedlings embryonic characteristics and to induce embryo-like structures from vegetative organs in *Arabidopsis* [4]. These results indicated the ability of *LEC1* to induce vegetative- to-embryonic transition. *LEC1* over expression caused accumulation of seed-specific storage protein and oil body protein in vegetative tissues [4]. Fatty acid biosynthetic genes were globally up-regulated in *LEC1* overexpressor [5]. The role of *LEC1* maintaining embryonic characteristics in vegetative organs requires auxin and sugars. The phenotype of *Arabidopsis tnp* mutant, a gain-of-function mutant of *LEC1*, could be strengthened with exogenous auxin and sugars [6]. *LEC1-LIKE* (*L1L*) that shows sequence similarity with *LEC1*, is required for normal embryogenesis. Although *L1L* and *LEC1* play different roles during embryo development, ectopic expression of *L1L* could rescue the defect of *lec1* mutant [7].

Another member of *LEC* genes, *LEC2*, encodes B3 domain transcription factors which are unique to plants. The *lec2* mutation caused pleiotropic defects in embryo development [3]. Ectopic expression of *LEC2* caused accumulation of lipid and seed storage protein in transgenic seedlings [8]. A number of genes regulated by *LEC2* were identified, providing information about the role of *LEC2* in somatic embryogenesis [9]. Auxin biosynthesis genes *YUC2* and *YUC4* can be activated by *LEC2* [10]. The capacity of somatic embryogenesis in *lec1lec2* double mutants was very low even in the presence of auxin. This suggested that formation of somatic embryo by auxin needs the function of *LEC* genes [11].

In addition, many genes such as *SERK*, *AGL15*, *BBM*, *WUS* and *PKL* are involved in somatic embryogenesis [1]. To gain insights into the mechanism by which *LEC* genes induce somatic embryogenesis, we ectopically expressed *AtLEC* gene in tobacco using inducible chimeric 35S:*AtLEC1*/*AtLEC2*-GR fusion construct. The results showed that ectopic expression of *LEC1* and *LEC2* could confer embryonic characteristics to transgenic tobacco seedling. Somatic embryogenesis and plant regeneration was occurred in a high frequency from *LEC2* transgenic seedlings grown in medium without application of exogenous plant growth regulators. However, regenerated plants were not obtained from *LEC1* overexpressors under the same culture condition. Ectopic expression of *LEC2* activated expression of globulin, oleosin, caleosin and LEA protein genes that normally expressed predominantly in maturation seeds. Genes encoding regulators that play important roles in embryo development such as *MADS-box protein 9*, *SERK1* and *leafy cotyledon 1-like* (*L1L*) were activated in the transgenic plants. These results indicated that *Arabidopsis LEC* genes could activate somatic embryogenesis process in transgenic tobacco plants, albeit to different extents.

## Results

### Determination of DEX Concentration

When testing the optimal concentration of DEX, we tested 5, 10, 20, 30, 40, 50 µM of DEX for embryonic callus induction and seedling regeneration. The result showed that 5–10 µM DEX induction for 20 days could not induce 100% callus formation in *LEC2* overexpressors. Induction with 20–50 µM DEX for 20 days could induce 100% callus formation with different size and quality. 20 µM DEX induced small size callus, while 30 µM DEX induced larger and better quality embryonic callus. Higher concentration (40–50 µM) of DEX could induce 100% callus formation. However, the regeneration rate of these calli decreased and the regeneration time increased. Therefore, 20 µM DEX is found to be most appropriate concentration to regenerate plant in a short time. To get more somatic embryos and later more regenerated seedlings from each callus, 30 µM DEX is found to be the optimal condition. In the digital gene expression experiment, we aim to analysis the ability of *LEC2* gene on somatic embryo induction, 30 µM DEX which could induce large amount of good quality embryonic callus was selected. Callus formation was not observed when *LEC1* transgenic seeds germinated on medium containing different concentration of DEX for 20 days.

### Ectopic LEC1 Expression Induced the Start of Embryonic Transition

**Figure 1:**
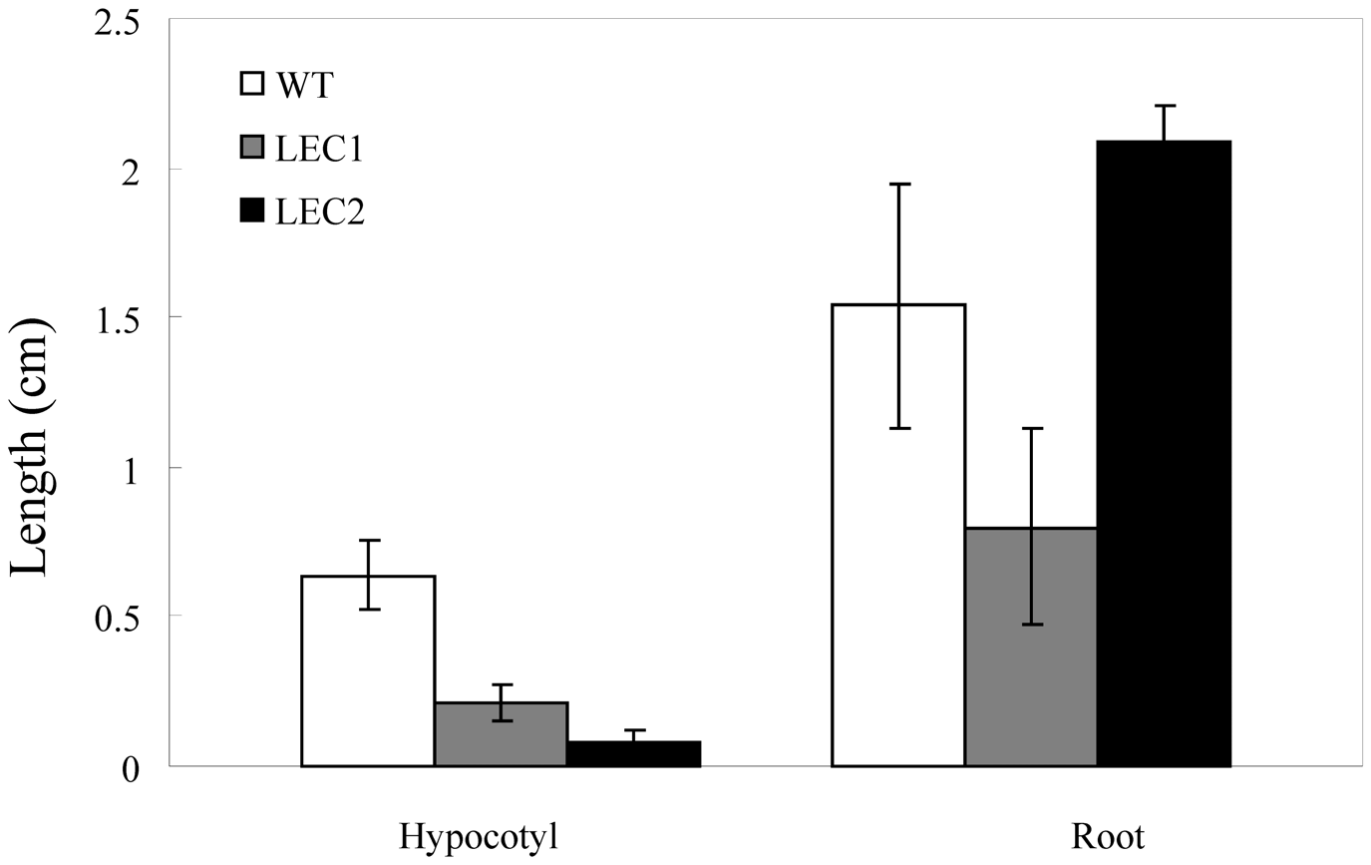
Hypocotyl and root length of transgenic and WT tobacco germinated on 30 µM DEX containing medium for 20 d. The *LEC1* transgenic seedling roots were shorter, but the *LEC2*’s were longer than the control.

**Figure 2:**
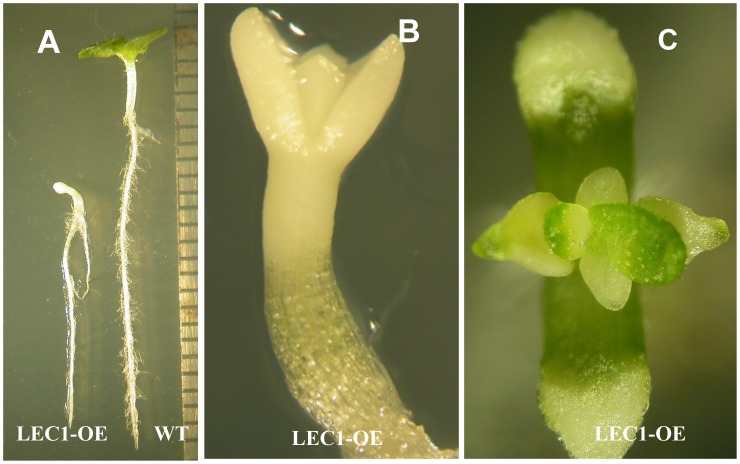
*LEC1* transgenic and WT tobacco seeds germinated on 30 µM DEX medium. (**A, B**): comparison of *LEC1* transgenic and WT tobacco seedlings on DEX medium for 12 d. (**C**) *LEC1* transgenic seedlings grown on 30 µM DEX medium for 25 d.

A 35S:*AtLEC1*-GR construct was introduced into tobacco plant by *Agrobacteria* mediated transformation. On MS medium containing 30 µM DEX, the *LEC1* transgenic homozygote seeds germinated two days later than WT controls. After 20 days induction, the roots and hypocotyls of transgenic seedling were shorter than the control (Figure 1). The transgenic seedlings exhibited embryonic characteristics such as the ivory and fleshy appearance of the whole plant and unexpanded cotyledons (Figure 2A, 2B). After growing on the DEX containing induction medium for 20–25 days, the transgenic seedlings produced fleshy and thick true leaves with pale color, some of which were green on the tip region of the leaf (Figure 2C). After the seedling grown on the 30 µM DEX medium for 40 days (during this period medium was not changed), 33% (43/131) plants produce true leaves. There was no obvious callus formation on the seedlings. The results indicated that *AtLEC1* could induce the start of embryonic transition but was not sufficient to form somatic embryos in tobacco seedlings after induction on 30 µM DEX medium for 40 days.

### Ectopic Expression of *LEC2* Induced Somatic Embryo Formation and Plant Regeneration

**Figure 3:**
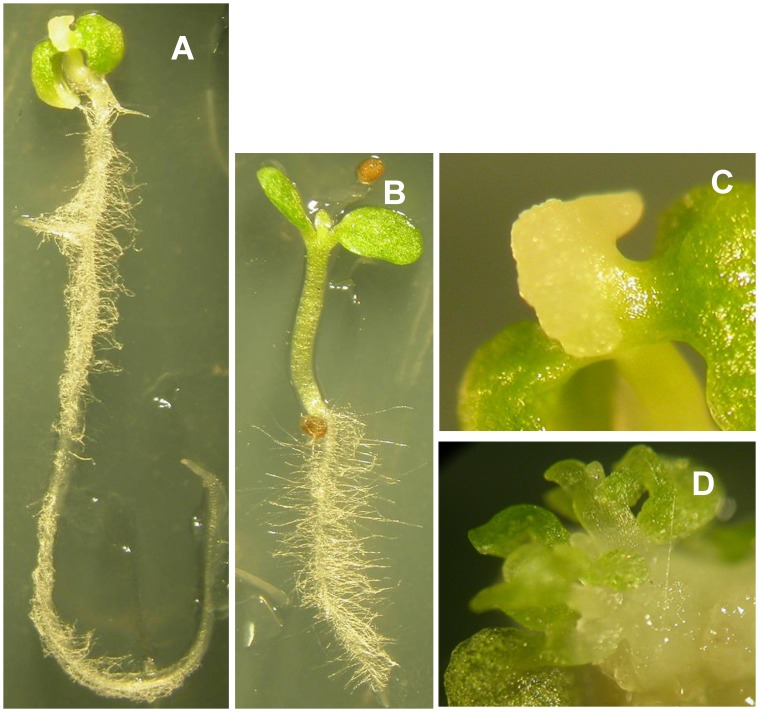
*LEC2* transgenic and WT tobacco seeds germinated on 30 µM DEX medium. (**A, B**) *LEC2* transgenic and wild type seedlings grown on 30 µM DEX medium for 20 d. (**C**) the close view of the callus in A. (**D**) shoot formation from callus of *LEC2* transgenic seedling grown on 30 µM DEX medium for 30 d.

A 35S:*AtLEC2*-GR construct was transformed into tobacco plants by *Agrobacteria*. When the homozygote transgenic seeds germinated on medium containing 30 µM DEX for 20 days the cotyledons could not expand and embryonic callus was induced on the shoot apical meristem (Figure 3A, 3C). The roots of the transgenic seedlings were much longer than the root of the wild type control (Figure 1, 3). However, the hypocotyls of the transgenic plants were found to be shorter (Figure 1, 3). After growing on DEX containing medium for about 40 days, somatic embryo-like structures emerged from the callus (Figure 3D). The results suggested that *AtLEC2* was sufficient to induce somatic embryo development in transgenic tobacco plants.

After transgenic seeds were germinated and grown in MS medium containing 10, 20, 30, 40 or 50 µM of DEX for 20 days, the embryonic calli were transferred to MS medium without DEX and exogenous plant hormone (MS_0_) for plant regeneration. About 200 pieces of callus for each DEX treatment were used in this experiment. Results showed that low DEX induced callus exhibited higher regeneration rate in MS_0_ medium than high DEX induced callus. Each callus induced by 10 µM of DEX could generate 5–10 plantlets on average and the regeneration rate was 100%. When DEX concentration was 30 µM, although the callus formation rate was 100%, plant regeneration rate was decreased to about 90% when transferred to MS_0_ medium. Results suggested that too high concentration of DEX should be avoided during callus induction period in order to obtain high and efficient regeneration of plants from the callus.

### Digital Gene Expression Profiling Analysis

To understand the mechanisms by which *LEC2* gene promote somatic embryo formation, we searched for genes affected by *LEC2* ectopic expression using digital gene expression profiling. As described above, 30 µM DEX could induce large amount of high quality embryonic callus. In the digital gene expression experiment, 30 µM DEX was selected for embryonic callus induction. High throughput sequencing generated about 12 million short reads, among which 5298 and 5568 unigenes were up- and down-regulated in *LEC2* transgenic tobacco (Table S1).

Genes normally expressed in embryo maturation processes are induced by ectopic *LEC2* activity. For example, genes encoding seed storage proteins include 7S and 11S globulin, vicilin, oleosin, caleosin and late embryogenesis abundant (LEA) protein were activated in *LEC2* transgenic seedlings. Most of genes acting in fatty acid and steroid biosynthesis were up-regulated in *LEC2* transgenic lines. These results demonstrated that *LEC2* regulates genes involved in seed maturation processes. The expression of sucrose synthase 2 (*SUS2*) that is involved in sugar accumulation was up-regulated in *LEC2* transgenic tobacco.

Key regulators in embryo development are activated by ectopic *LEC2* activity. Regulatory genes including *MADS-box protein 9, L1L, SERK1* were activated in *LEC2* transgenic seedlings (2 x). Interestingly, the expression of *L1L* gene was up-regulated for more than one hundred-fold in *LEC2* transgenic lines. Some plant-specific transcription factors, including members of the *NAC* and GRAS gene families were up-regulated.

In *LEC2* transgenic tobacco, expression of *ARF8* and *ARF5* were up-regulated just like in *LEC1* transgenic plants (data not shown). Auxin efflux facilitator *PIN1* and *PIN2* that mediate auxin polar transport were up-regulated by *LEC2*. The gene encoding IAA13 protein was down-regulated in *LEC2* transgenic seedlings. *ARF10* known to control cell division and cell differentiation in root cap and required for root cap development was up-regulated [12]. Most of genes in ethylene signaling pathway were down-regulated. *CKX* (cytokinin oxidase/dehydrogenase) that could reduce endogenous cytokinin content when ectopically expressed [13], was up-regulated in *LEC2* transgenic seedlings. *LEC2* repressed expression of *GA3ox2*. This is consistent with result of previous study [14]. While *GA20ox*, one of the GA biosynthesis genes, was up-regulated, expressions of GA2ox and DELLA genes were found to be down-regulated.

Up- or down-regulation of genes involved in ABA signaling was observed. *ABI* (*Abscisic Acid Insensitive*) gene, a positive regulator in ABA signaling, was up-regulated in *LEC2* transgenic tobacco. In *LEC2* transgenic seedlings, many genes involved in photosynthesis and biosynthesis of flavonoids were down-regulated, which was coincident with the pale color and low chlorophyll content of the transgenic plants. DICER like protein and ARGONAUTE are key enzymes in miRNA biosynthesis which is crucial in embryogenesis. The expression of genes encoding these enzymes was up-regulated in *LEC2* transgenic lines.

## Discussion

Over expression of *AtLEC1* and *AtLEC2* genes was sufficient to induce somatic embryogenesis in *Arabidopsis*. Here we showed that expression of *AtLEC1* and *AtLEC2* in tobacco could activate somatic embryogenesis process, although in a different extend. Cotyledons of *LEC1* transgenic tobacco were ivory and fleshy and could not expand; their hypocotyls were stubby. These results suggest that *AtLEC1* can help start the transition from vegetative growth to somatic embryogenesis, but is not sufficient to complete this process in tobacco after 20 days of induction. A previous study has shown that constitutive expression of *LEC1* in *lec1* background by 35S promoter could induce somatic embryo formation in few transgenic lines [4]. In our experiment, when *LEC1* seedlings grown on medium containing 30 µM DEX for 40 days (without changing medium), most of the plants produced true leaves. The recovery of vegetative growth may be due to exhaust DEX in the medium and resulted in the cessation of *LEC1* expression. Whether longer time induction on DEX medium could induce somatic embryogenesis in *LEC1* transgenic tobacco is unknown. The shoot apical meristem of *LEC2* transgenic plants formed callus and no true leaf could be produced on DEX containing medium. When callus was transferred to MS_0_ medium, plantlets were regenerated from each callus. These results were in agreement with the previous report [8]. Similarities and differences in the phenotype of ectopic expression of *LEC1* and *LEC2* in *Arabidopsis* and tobacco indicated their partially overlapping but not identical roles in somatic embryo induction between *LEC1* and *LEC2*, as well as in different species. Our results showed that *LEC2* could be a better candidate for improving the regeneration ability of crop plants.

Studies showed that *LEC1* and *LEC2* cause accumulation of seed storage reserves and *LEC2* directly induce genes involved in maturation processes before formation of somatic embryos [4], [8], [9], [10]. Our study proved that many genes in seed maturation phase are activated by ectopic expression of *LEC2* in tobacco. Many genes involved in biosynthesis of fatty acid and steroid, were also up-regulated, consistent with the fact that *LEC1* and *LEC2* increase fatty acid and lipid accumulation [5], [15]. *LEC2* induced the expression of *SUS2*, a sucrose synthase gene, in maturation phase. Taken together, our results suggested that ectopic expression of *AtLEC2* induces maturation processes in transgenic tobacco.

Our results showed that many regulatory factors involved in embryo development and somatic embryogenesis, including *MADS-box protein 9*, *L1L*, and *SERK1*, were activated by *LEC2*. *MADS9* belonging to *AGL15* subgroup of the MADS box family, expressed mainly during embryogenesis [16], [17]. Constitutive expression of *AGL15* enhances competence of somatic embryo formation from the shoot apical meristems [18]. Braybrook [9] showed that *LEC2* could induce the expression of *FUSCA3* and *ABI3* and could directly regulate *AGL15*. *LEC2*, *FUS3* and *ABI3* were found as direct target genes of *AGL15* [19]. Our results suggested that *LEC2* activated *MADS9*, one member of the *AGL15* subgroup. However, expression changes of *FUS3* and *ABI3* were not observed in *LEC2* transgenic tobacco. *LEC2* might regulate somatic embryogenesis through *FUS3* and *ABI3* indirectly in ways which remains unknown. *L1L* plays distinct roles from *LEC1* but over expression of *L1L* could rescue *lec1* mutant. Ectopic expression of *LEC2* increase expression of *LEC1*, and *LEC2* could be induced by *LEC1* [10], [16], [20]. Our results demonstrated that ectopic expression of *LEC2* could up-regulate *L1L* in transgenic tobacco. It was suggested that *L1L* may play key roles in *LEC2* inducing somatic embryogenesis. SERK1 is a key factor promoting vegetative-to-embryonic transition and over expression of *SERK1* increases somatic formation. SERK1 was considered as a marker of embryonic cells [21]. Up-regulation of *SERK1* in *LEC2* transgenic tobacco provided further evidence for its roles in promoting embryogenesis.

*LEC2* activated several transcription factors including *NAC*, *AP2* and *GRAS* gene family. NAC proteins play diverse roles in a wide range of plant developmental processes, such as embryo development, shoot apical meristem development [22], [23], lateral root development [24], and hormone signaling [24], [25], [26]. HAM (hairy meristem), a member of GRAS family, regulates both shoot and root meristems [27]. Our results strongly suggested that formation of embryonic callus from the shoot apical meristem and hairy phenotype of the callus in *LEC2* transgenic tobacco seedlings was correlated with up-regulation of *HAM*.

Hormones play key roles in embryo development and somatic embryogenesis. Braybrook [9] showed that *LEC2* activated gene expression of IAA30, one of auxin signaling proteins, which may affect plant response to auxin or confer competency for somatic embryogenesis. Stone [10] proved that *LEC2* also induced genes involved in auxin biosynthesis such as *YUC2*, *YUC4*, *IAA1*, *IAA17* and *ACS4*. Our study showed that both *PIN1* and *PIN2* were induced in *LEC2* transgenic tobacco. Auxin-responsive genes, including *ARF3*, *ARF5* and *ARF8*, play diverse roles in reproductive organ and embryo developmental processes [28], [29], [30], [31], [32]. We found that *ARF5, ARF8* and *ARF10* were activated by *LEC2*. *IAA13*, a negative regulator in auxin signaling, was down-regulated. *ARF19* which can be induced by IAA or ethylene treatment [33] was down-regulated. Roustan [34] showed that inhibition of ethylene production can increase somatic embryogenesis. Genes involved in ethylene biosynthesis and response were down-regulated in super-embryogenic line 2HA compared with the non-embryogenic progenitor [35]. Somatic embryogenesis was enhanced by AgNO3, an ethylene inhibitor, in several plant species [36]. Zheng [37] showed that expression of some genes involved in ethylene signaling pathway and that ethylene production was increased in the process of *GmAGL15* promoting somatic embryogenesis in soybean. Our results showed that most genes involved in ethylene signaling pathway were down-regulated by *LEC2*. *CKX* expression could lead to more root branches and larger root meristem [14]. Up-regulation of *ARF10* and *CKX* was consistent with the densely grown hairy structure on embryonic callus and longer roots of *LEC2* overexpressor. *MYC2*, a positive regulator in JA signaling, was down-regulated. Chen [38] showed that *MYC2* directly represses expression of *PLT1* and *PLT2* which are important transcription factors in auxin signaling pathways. Previous studies indicated that reduced levels of GA induced somatic embryo formation [39] and that *LEC2* repressed the expression of GA biosynthesis gene *GA3ox2* [14]. In our study, over expressing *LEC2* reduced the expression of *GA3ox2*, however, the gene expression of GA inactive enzyme GA2ox and the GA signaling negative factor DELLA was also down-regulated in transgenic tobacco. Several members of the ABI family are key transcriptional factors that regulate late embryogenesis and seed maturation [40], [41], [42], [43]. *ABI* gene was up-regulated in *LEC2* transgenic tobacco.

*LEC1* and *LEC2* could repress anthocyanin accumulation, trichomes formation and induce chlorophyll degradation and desiccation tolerance through activation of *FUS3* and *ABI3* [44]. We found that genes involved in biosynthesis of anthocyanin, chlorophyll and genes in photosynthesis were down-regulated in transgenic seedlings. However, we did not detect any significant changes in *FUS3* in the transgenic tobacco plants. Taken together, *AtLEC2* could initiate the transition from vegetative growth to embryogenesis by affecting the expression of key transcription factor genes, and genes involved in hormone biosynthesis and signaling.

## Materials and Methods

### Vector Construction and Gene Transformation

Binary vector containing 35S:*AtLEC2*-GR was provided by Harada’s lab (University of California, Davis). To make 35S:*AtLEC1*-GR, the inducible expression element of 35S:*AtLEC2*-GR was amplified, cloned into pGEM-T EASY (promega). The ORF region of the *AtLEC1* cDNA clone was amplified with *LEC1 Xho*I (5'-TATACTCGAGATGGAACGTGGCGCACC-3') and *LEC1 ClaI* (5'-CCATCGAT TTCTTATACTGACC-3') primers and was used to replace the *AtLEC2* gene in pGEM-T EASY vector and generated pGEM-*AtLEC1*-GR construct. The inducible expression element 35S:*AtLEC1*-GR was recombined into pCAMBIA2300 and binary vector containing 35S:*AtLEC1*-GR was constructed. Constructs were transferred into *Agrobacterium tumefaciens* (LBA4404) and transformed into *Nicotiana tabacum* cv SR1 using leaf discs method [45]. *LEC1* and *LEC2* transgenic tobacco were selected on MS medium [46] containing 150 mg L^−1^ of kanamycin or 4 mg L^−1^ Basta, respectively.

### Callus Induction and Shoot Regeneration from Transgenic Seedlings

Homozygote transgenic seeds were surface sterilized and germinated on MS medium containing 5, 10, 20, 30, 40, 50 µM of DEX for 20 days to determine the optimal concentration for callus induction. Callus from *LEC2* seedlings (35S:*AtLEC2*-GR), seedlings with callus or seedlings without callus (35S:*AtLEC1*-GR) were transferred to MS_0_ medium (no DEX and exogenous hormone was supplied) for shoot regeneration.

### RNA Extraction

RNA was extracted from transgenic and wild-type tobacco seedlings. Two hundred milligram of tissue was ground with a pestle and mortar in liquid nitrogen. Powder was extracted with 600 µl 65°C CTAB [0.1 M Tris-HCl (pH 8.0), 25 mM EDTA (pH 8.0), 2 M NaCl, 2% CTAB, 2% PVP-40]. The aqueous phase was extracted twice with an equal volume of chloroform/phenol (1∶1 vol/vol) and chloroform. RNA was precipitated by mixing with 1/3 volume of 8 M LiCl at 4°C overnight. After centrifugation the precipitated RNA were washed twice with 70% alcohol and dissolved in diethyl pyrocarbonate-treated water. Total RNA was analyzed by electrophoresis using 1% agarose gel.

### High Throughput Sequencing

We isolated total RNA from 35S:*AtLEC2*-GR transgenic tobacco grown for 20 days on MS medium containing 30 µM DEX. Total RNA from wild typed tobacco seedlings grown for 20 days on MS medium containing 30 µM DEX were used as controls. The quality and quantity of the purified RNA from each sample was determined by Agilent 2100. Beads with Oligo (dT) were used to enrich polyA mRNA. mRNAs were interrupted to short fragment (200–700 nt) using fragmentation buffer. These short mRNA fragments were used as templates to synthesize the first strand cDNA using random hexamers. The second strand cDNA was synthesized using DNA polymerase I (New England Biolabs), RNase H (Invitrogen), dNTPs and buffer. The cDNA were purified by QiaQuick PCR kit and then carried through end repair, polyA tails and adaptors were added up. Fragments with suitable size were recovered through agarose gel electrophoresis and amplified by PCR. The PCR products were sequenced using Illumina HiSeq™2000.

### Digital Gene Expression Profile Analysis

Raw reads were acquired through sequencing and clean reads were obtained by removing the impure sequences. The clean reads were mapped to reference sequences using software SOAPaligner/soap2 according to the criteria that no more than 2 bases mismatches were allowed in the alignment. The URL of reference sequences is from http://www.pngg.org/tgi/login-new.html. After alignment, a series of statistical and bioinformatical analysis were followed. The quality of sequencing was evaluated through clean reads percentage, sequencing saturation, reads distribution analysis. Gene expression level is calculated by the numbers of reads mapped to the reference sequences. The data was normalized to RPKM (Reads Per Kb Per Million Reads) with the following formula: PPKM = [106C/(NL/103)]. Supposing RPKM (A) to be the expression of gene A, C is the number of reads that uniquely aligned to gene A. N is the total number of reads that uniquely aligned to all genes. L is the number of bases of gene A. After screening of differentially expressed genes (DEGs), GO function analysis and KEGG pathway analysis were carried out. Gene expression was considered up-regulated or down-regulated whose value of log2 Ratio (LEC/WT) ≥1.

## Supporting Information

Table S1 **Genes that were affected by** ***AtLEC2*** **ectopic expression using digital gene expression profiling analysis.**
(XLS)
